# Clinical Holistic Medicine (Mindful, Short-Term Psychodynamic Psychotherapy Complemented with Bodywork) in the Treatment of Experienced Impaired Sexual Functioning

**DOI:** 10.1100/tsw.2007.70

**Published:** 2007-03-02

**Authors:** Søren Ventegodt, Suzette Thegler, Tove Andreasen, Flemming Struve, Lars Enevoldsen, Laila Bassaine, Margrethe Torp, Joav Merrick

**Affiliations:** ^1^ Quality of Life Research Center, Teglgårdstræde 4-8, DK-1452 Copenhagen K, Denmark; ^2^ Research Clinic for Holistic Medicine, Copenhagen, Denmark; ^3^ Nordic School of Holistic Medicine, Copenhagen, Denmark; ^4^ Scandinavian Foundation for Holistic Medicine, Sandvika, Norway; ^5^ Interuniversity College, Graz, Austria; ^6^ Zusman Child Development Center, Soroka University Medical Center, Ben Gurion University of the Negev, Beer-Sheva, Israel; ^7^ National Institute of Child Health and Human Development, Jerusalem, Israel; ^8^ Office of the Medical Director, Division for Mental Retardation, Ministry of Social Affairs, Jerusalem, Israel

**Keywords:** human development, quality of life, sexual dysfunction, psychodynamic therapy, body work, Denmark, short-term psychodynamic psychotherapy (STPP), holistic medicine, existential healing, salutogenesis, Antonovsky

## Abstract

In this clinical follow-up study, we examined the effect of clinical holistic medicine (psychodynamic short-term therapy complemented with bodywork) on patients with poor self-assessed sexual functioning and found that this problem could be solved in 41.67% of the patients ((95% CI: 27.61—56.7%; 1.75 < NNT < 3.62, p = 0.05). The bodywork was inspired by the Marion Rosen method and helped the patients to confront painful emotions from childhood trauma(s), and thus accelerated and deepened the therapy. The goal of therapy was the healing of the whole life of the patient through Antonovsky-salutogenesis. In this process, rehabilitation of the character and purpose of life of the patient was essential, and assisted the patient to recover his or her sense of coherence (existential coherence). We conclude that clinical holistic medicine is the treatment of choice if the patient is ready to explore and assume responsibility for his or her existence (true self), and willing to struggle emotionally in the therapy to reach this important goal. When the patient heals existentially, quality of life, health, and ability to function in general are improved at the same time. The therapy was “mindful” in its focus on existential and spiritual issues. The patients received in average 14.8 sessions at the cost of 1,188 EURO.

## INTRODUCTION

About 25–50% of the western population complains about sexual issues[1] and with more specific questions asked, the larger the group of patients that have such problems becomes. In our clinical work, we measured sexual ability in 109 patients who entered the Research Clinic for Holistic Medicine during the 2004–2005 period and found that 48 of these patients complained about significant sexual issues regarding their self-assessed ability to function sexually. These patients entered a treatment process where their sexual ability could be addressed through rehabilitation of their natural being and knowledge of self. The intention was to heal their whole life through induction of Antonovsky-salutogenesis[2,3]. In this paper, we analyze the effect that our treatment had on the patients who presented with self-assessed sexual problems.

## METHOD

The patients were included in this study if they assessed their own sexual functioning (each patient received a questionnaire before start of treatment) as impaired or very impaired before treatment started. They received treatment according to clinical holistic medicine[4,5,6], a kind of psychodynamic short-term therapy earlier found effective for a long list of health problems[7,8]. The patients were also evaluated for sexual issues that existed along three axis: desire, orgasmic dysfunction, and sexually related pain (mostly pain during intercourse, primary vulvodynia, or pelvic tension pain)[9]. The bodywork was inspired by Marion Rosen and helped the patients to confront old emotional pain from childhood trauma repressed to the body-mind[10].

## RESULTS

Forty-eight patients entered the study having self-assessed impaired ability to function sexually before treatment (self-assessed as being “impaired” or “very impaired”). Twenty patients rated their sexual functioning as adequate after treatment: (self-assessed sexual ability: very good, good, or neither good nor bad). Of those 20 patients, eight completely resolved their problem (rating good or very good) and 12 were improving (rating: neither good nor bad). Eleven of the patients continued to self-assess their sexual functioning as impaired after the treatment (self-assessed sexual ability: bad or very bad). The response rate of follow-up survey 1 year after was 64.6%. Seventeen patients were classified as nonresponders on follow-up. After the treatment, 28 patients were either still poorly functioning sexually or classified as nonresponders on follow-up, or withdrew from the study early.

The “rate of cure” of the treatment was 20/48 = 41.7% (95%CI: 27.6–57.0%) (Table 1)[11]. Number Needed to Treat (NNT) of clinical holistic medicine with sexually poorly functioning patients = 1.75–3.62. Number Needed to Harm (NNH) was estimated from treating more than 500 patients in our clinic since the year 2000 with this therapy and none of which had severe side effects or harmed themselves or other people during the therapy; NNH estimated >500. The patients healed not only their sexuality, but also their whole being because of the induction of Antonovsky-salutogenesis. Both physical and mental health; relations to self, friends, and partner; and ability to function socially and to work was improved, as was the self-assessed quality of life. Quality of life, health, and relations were measured with QOL1 and QOL5[6,12].

Table 2 shows that 43 patients had sexual issues related to desire, 16 patients had problems related to sexually related pain, 24 patients suffered from orgasmic dysfunction, and 33 patients had other sexual problems. Patients could have more than one problem. Interestingly, physical health; mental health; relation to self, friends, and partner; ability to love, function socially, and work (meaning ability to sustain full-time work); and self-evaluated quality of life by QOL1[12] did also improve for many of the patients during the therapy. The general beneficial effect of the therapy is due to the induction of Antonovsky-salutogenesis[2,3].

Fifty-six percent of the clinic's patients reported sexual problems and received, on average, 14.8 sessions at the cost of 1,188 EURO. Tables 3–6 show that when the patient with the experience of sexual inadequacy healed her/his life (enters the state of salutogenesis), the sexual issues were resolved and all other dimensions of existence were improved as well.

**TABLE 1**
**Patients with Severe Sexual Problems Who Entered the Study**

| | Before Treatment | After Treatment |
|---|---|---|
| Low self-assessed sexual ability | 48 | 11 |
| High ability to function sexually | 0 | 20 = 41.67% (95% CI: 27.61–56.7%) |
| Nonresponders or dropouts | — | 17 |
| Low sexual ability, nonresponder or dropout | — | 28 |

**TABLE 2**
**Summary of Patient Identified Sexual Issues**

| | | Self-Evaluated Physical Health | | | | Self-Evaluated Mental Health | | | | Relation to Myself | | | | Relation to Friends | | | |
|---|---|---|---|---|---|---|---|---|---|---|---|---|---|---|---|---|---|
| | | Before | After | Δ | p | Before | After | Δ | p | Before | After | Δ | p | Before | After | Δ | p |
| Desire | Val | 2.7 | 2.33 | 0.37 | .019 | 3.65 | 2.42 | 1.23 | .000 | 3.28 | 2.41 | 0.87 | .000 | 2.61 | 2.18 | 0.43 | .011 |
| | N | 43 | 33 | 10 | | 43 | 33 | 10 | | 43 | 34 | 9 | | 43 | 34 | 9 | |
| Pain | Val | 3 | 2.4 | 0.6 | NS | 3.25 | 2.4 | 0.85 | NS | 3.19 | 2.2 | 0.99 | .032 | 2 | 1.5 | 0.5 | NS |
| | N | 16 | 10 | 6 | | 16 | 10 | 6 | | 16 | 10 | 6 | | 16 | 10 | 6 | |
| Orgasmic dysfunction | Val | 2.75 | 2.35 | 0.4 | .016 | 3.17 | 2.29 | 0.88 | .000 | 3.04 | 2.39 | 0.65 | .006 | 2 | 1.89 | 0.11 | NS |
| | N | 24 | 17 | 7 | | 24 | 17 | 7 | | 24 | 18 | 6 | | 24 | 18 | 6 | |
| Other problems | Val | 2.76 | 2.21 | 0.55 | NS | 3.55 | 2.63 | 0.92 | .002 | 3.24 | 2.36 | 0.88 | .001 | 2.61 | 2.04 | NS | .012 |
| | N | 33 | 24 | 9 | | 33 | 24 | 9 | | 33 | 24 | 9 | | 33 | 24 | 9 | |

| | | Relation to Your Partner | | | | Self-Evaluated Ability to Love | | | | Self-Evaluated Sexual Function | | | |
|---|---|---|---|---|---|---|---|---|---|---|---|---|---|
| | | Before | After | Δ | p | Before | After | Δ | p | Before | After | Δ | p |
| Desire | Val | 5 | 3.88 | 1.12 | .016 | | 2.5 | 0.86 | .001 | 3.35 | 2.71 | 0.64 | .014 |
| | N | 43 | 34 | 9 | | 42 | 34 | 8 | | 43 | 34 | 9 | |
| Pain | Val | 4.3 | 2.9 | 1.4 | NS | 3.31 | 1.9 | 1.41 | .011 | 3.94 | 3 | 0.94 | .004 |
| | N | 16 | 10 | 6 | | 16 | 10 | 6 | | 16 | 10 | 6 | |
| Orgasmic dysfunction | Val | 4 | 3.11 | 0.89 | NS | 3 | 2.44 | 0.56 | NS | 3.21 | 2.78 | 0.43 | NS |
| | N | 24 | 18 | 6 | | 24 | 18 | 6 | | 24 | 18 | 6 | |
| Other problems | Val | 4.72 | 3.46 | 1.26 | NS | 3.39 | 2.29 | 1.1 | .002 | 3.46 | 2.67 | 0.79 | .029 |
| | N | 32 | 24 | 8 | | 33 | 24 | 9 | | 33 | 24 | 9 | |

| | | Self-Evaluated Social Function | | | | Self-Evaluated Working Capacity | | | | Self-Evaluated Quality of Life (QOL1 | | | |
|---|---|---|---|---|---|---|---|---|---|---|---|---|---|
| | | Before | After | Δ | p | Before | After | Δ | p | Before | After | Δ | p |
| Desire | Val | 2.79 | 2.18 | 0.61 | .002 | 3.09 | 2.53 | 0.56 | .025 | 3.44 | 2.32 | 1.12 | .000 |
| | N | 43 | 34 | 9 | | 43 | 34 | 9 | | 43 | 34 | 9 | |
| Pain | Val | 2.63 | 1.8 | 0.83 | .025 | 3.38 | 2.4 | 0.98 | .037 | 3.31 | 2.3 | 1.01 | .029 |
| | N | 16 | 10 | 6 | | 16 | 10 | 6 | | 16 | 10 | 6 | |
| Orgasmic dysfunction | Val | 2.42 | 2.17 | 0.25 | NS | 3 | 2.67 | 0.33 | NS | 3.46 | 2.61 | 0.85 | .000 |
| | N | 24 | 18 | 6 | | 24 | 18 | 6 | | 24 | 18 | 6 | |
| Other problems | Val | 3.06 | 2.29 | 0.77 | .001 | 3.06 | 2.46 | 0.6 | NS | 3.36 | 2.33 | 1.03 | .001 |
| | N | 33 | 24 | 9 | | 33 | 24 | 9 | | 33 | 24 | 9 | |

## DISCUSSION

Sexual issues are very common and often related to existential and mental problems. In spite of good medical advice and sexological training programs, most patients with a compromised ability to function sexually continue to have sexual problems, which can continue even for years. We have learned from working with many patients during the last 6 years that even severe sexual inadequacy, both male and female, is often due to existential factors, such as not knowing one's self sufficiently. Sexual problems are not only connected to physical or mental factors, and this is why psychotherapy alone often is not able to solve sexual problems, while holistic existential therapy combined with bodywork often is.

**TABLE 3**
**Patients with Sexual Problems who Succeeded in Experiencing Antonovsky-Salutogenesis (T-Test Paired Samples Statistics)**

| | | Mean | N | Std. Deviation | Std. Error Mean |
|---|---|---|---|---|---|
| Physical health | Before | 2.7368 | 19 | 0.87191 | –0.20003 |
| | After | 2.2105 | 19 | 0.85498 | 0.19615 |
| Mental health | Before | 3,7000 | 20 | 0.86450 | 0.19331 |
| | After | 2.1000 | 20 | –0.85224 | –0.19057 |
| Self-esteem | Before | 3.5000 | 20 | 0.76089 | 0.17014 |
| | After | 2.3500 | 20 | –0.98809 | 0.22094 |
| Relation to friends | Before | 2.5500 | 20 | 0.99868 | 0.22331 |
| | After | 1.9500 | 20 | 0.88704 | 0.19835 |
| Relation to partner | Before | 4.2000 | 20 | 2.01573 | 0.45073 |
| | After | 2.6500 | 20 | 2.05900 | 0.46041 |
| Ability to love | Before | 3.6500 | 20 | 1.18210 | 0.26433 |
| | After | 2.1500 | 20 | 1.26803 | 0.28354 |
| Sexual ability | Before | 4.4000 | 20 | 0.50262 | 0.11239 |
| | After | 2.5000 | 20 | 0.68825 | 0.15390 |
| Social ability | Before | 3.0500 | 20 | 1.14593 | 0.25624 |
| | After | 2.0000 | 20 | 0.79472 | 0.17770 |
| Work ability | Before | 3.1053 | 19 | 0.93659 | 0.21487 |
| | After | 2.1579 | 19 | 1.06787 | 0.24499 |
| Quality of life | Before | 3.6000 | 20 | 0.99472 | 0.22243 |
| | After | 2.2500 | 20 | 1.01955 | 0.22798 |

A successful strategy to solving sexual problems of general sexual inadequacy seems to be personal development of the sense of coherence, healing the whole being, not only of sexual life. The combination of psychodynamic therapy and bodywork helps the patient to confront the painful sexually related emotion in an efficient way and creates fast, affordable, and lasting results with no side effects.

In the course of the treatment, the patient must be willing to face deep existential problems and often very unpleasant feelings, such as shame, guilt, and hopelessness, when the often very early traumas are confronted, and the old emotional charge is reintegrated. The goal of the psychodynamic therapy is for the patients to learn to know their true selves. Not all patients are ready for that, so we believe that clinical holistic therapy is the therapy of choice when the patient is motivated for a deep, inner exploration.

**TABLE 4**
**Paired Samples Test**

| | Paired Differences | | | | | t | df | Significance (Two–Tailed) |
|---|---|---|---|---|---|---|---|---|
| | Mean | Std. Deviation | Std. Error Mean | 95% CI of Difference | | | | |
| | | | | Lower | Upper | | | |
| Physical health | 0.5263 | 0.84119 | 0.19298 | 0.1209 | 0.9318 | 2.727 | 18 | 0.014 |
| Mental health | 1.6000 | 1.18766 | 0.26557 | 1.0442 | 2.1558 | 6.025 | 19 | 0.000 |
| Self-esteem | 1.1500 | 1.18210 | 0.26433 | 0.5968 | 1.7032 | 4.351 | 19 | 0.000 |
| Relation to friends | 0.6000 | 1.18766 | 0.26557 | 0.0442 | 1.1558 | 2.259 | 19 | 0.036 |
| Relation to partner | 1.5500 | 2.06410 | 0.46155 | 0.5840 | 2.5160 | 3.358 | 19 | 0.003 |
| Ability to love | 1,5000 | 1.50438 | 0.33639 | 0.7959 | 2.2041 | 4.459 | 19 | 0.000 |
| Sexual ability | 1.9000 | 0.85224 | 0.19057 | 1.5011 | 2.2989 | 9.970 | 19 | 0.000 |
| Social ability | 1.0500 | 1.31689 | 0.29447 | 0.4337 | 1.6663 | 3.566 | 19 | 0.002 |
| Work ability | 0.9474 | 1.22355 | 0.28070 | 0.3576 | 1.5371 | 3.375 | 18 | 0.003 |
| Quality of life | 1.3500 | 1.34849 | 0.30153 | 0.7189 | 1.9811 | 4.477 | 19 | 0.000 |

**TABLE 5**
**Paired Samples Statistics**

| | | Mean | N | Std. Deviation | Std. Error Mean |
|---|---|---|---|---|---|
| Relations | Before | 3.4167 | 20 | 0.93580 | 0.20925 |
| | After | 2.3167 | 20 | 0.86839 | 0.19418 |
| Ability | Before | 3.5395 | 19 | 0.65226 | 0.14964 |
| | After | 2.1842 | 19 | 0.75389 | 0.17295 |
| QOL (QOL 5) | Before | 3.3509 | 19 | 0.64763 | 0.14858 |
| | After | 2.2456 | 19 | 0.68351 | 0.15681 |
| Health-QOL-Ability (QOL 10) | Before | 3.3735 | 18 | 0.59609 | 0.14050 |
| | After | 2.1574 | 18 | 0.58049 | 0.13682 |

## CONCLUSIONS

Clinical holistic medicine is our name for psychodynamic short-term therapy complemented with bodywork. The rehabilitation of character and purpose of life is essential, and assisting the patient to recover his or her existential coherence is the primary intent of the therapy. In this paper, we found that 41.67% (95% CI: 27.61–56.7%) were helped. The NNT of clinical holistic medicine with sexually poorly functioning patients = 1.75–3.62. The NNH was estimated as >500.

**TABLE 6**
**Paired Samples Test**

| | Paired Differences | | | | | t | df | Significance (Two–Tailed) |
|---|---|---|---|---|---|---|---|---|
| | Mean | Std. Deviation | Std. Error Mean | 95% CI of Difference | | | | |
| | | | | Lower | Upper | | | |
| Relations | 1.1000 | 0.99766 | 0.22308 | 0.6331 | 1.5669 | 4.931 | 19 | 0.000 |
| Ability | 1.3553 | 0.89488 | 0.20530 | 0.9239 | 1.7866 | 6.601 | 18 | 0.000 |
| QOL (QOL 5) | 1.1053 | 0.76419 | 0.17532 | 0.7369 | 1.4736 | 6.304 | 18 | 0.000 |
| Health-QOL-Ability (QOL 10) | 1.2160 | 0.65814 | 0.15512 | 0.8888 | 1.5433 | 7.839 | 17 | 0.000 |

## ACKNOWLEDGMENTS

This study was supported by grants from IMK Almene Fond. The quality of life research was originally approved by the Copenhagen Scientific Ethical Committee under number (KF)V.100.2123/91 and later correspondence.
